# Focused and automatic subtypes of skin picking disorder

**DOI:** 10.1017/S1092852925100667

**Published:** 2025-11-05

**Authors:** Austin Huang, Madison Collins, Jon E. Grant

**Affiliations:** Department of Psychiatry & Behavioral Neuroscience, University of Chicago, Pritzker School of Medicine, Chicago, IL, USA

**Keywords:** Skin picking disorder, excoriation, focused, automatic, cognition, comorbidity

## Abstract

**Objective:**

While prior studies have analyzed Skin Picking Disorder as a unitary condition, little research has been done examining clinical and neurocognitive characteristics of specific subtypes. The objective of this study is to analyze differences in impulsivity, emotional regulation, symptom severity, cognitive performance, and the presence of comorbid psychiatric conditions between focused and automatic subtypes of Skin Picking Disorder.

**Methods:**

83 adults aged 18–65 with skin picking disorder were enrolled at the University of Chicago and separated into 4 skin picking subtype groups based on high or low levels of focused and automatic picking scores on the Milwaukee Inventory for the Dimension of Adult Skin Picking. The 4 subtype groups were separated using K-means clustering. Each group completed the same clinical and neurocognitive assessments. ANOVA or Chi-Squared tests were used to analyze differences in assessment outcomes.

**Results:**

Higher focused picking scores were significantly associated with greater symptom severity and impairment. Differences in levels of automatic/focused picking were not associated with impulsivity, emotional/behavior regulations, or neurocognitive outcomes.

**Conclusions:**

The findings suggest that focused skin pickers are likely to have more impairment due to their behavior compared to automatic or mixed pickers; however, overall, the groups did not differ in clinical or neurocognitive measures. Thus, it is unclear whether focused and automatic picking are particularly useful clinically in subtyping skin picking disorder.

## Introduction

Skin picking disorder is a psychiatric disorder characterized by repetitive picking of skin, resulting in tissue damage that leads to significant distress or functional impairment.^1^ Past epidemiological studies have indicated that skin picking disorder has an estimated prevalence rate of 1.4%–.4%^2^ and has also been shown to be more common in females^3^ with onset usually in adolescence or young adulthood.^4^ Clinically, patients with skin picking disorder have reported elevated rates of comorbid depression, anxiety, and attention deficit hyperactivity disorder compared to the general population.^5^ Additionally, past neurocognitive analyses have suggested that people with skin picking disorder are more likely to have difficulties with impulsivity,^6^ emotional regulation,^7^ motor inhibition,^8^ and harm avoidance.^9^

Prior research has attempted to understand whether skin picking disorder may be better understood as having different phenomenological subtypes. Arnold et al.^10^ found that in semi-structured interviews of 34 adults with skin picking disorder, 24% reported being fully “aware” of their picking episodes while 76% reported picking outside of their awareness. Walther et al.^11^ identified an “automatic” and “focused” subtype, and created the Milwaukee Inventory for the Dimension of Adult Skin Picking (MIDAS), which has been a valid and reliable assessment in measuring the degree that individuals with skin picking disorder engage in “focused” picking (that is the individual is aware/conscious of their picking), “automatic” (that is the individual is unaware and picks habitually), or “mixed” (that is the individual exhibits both focused and automatic picking). Gallinat et al.^12^ found that visual/tactile cues and the desire to pick the skin were more important for the focused style, while boredom and concentration problems were more prominent in automatic skin picking.

Recent studies have suggested that the skin picking disorder subtypes may differ in personality traits and cognitive functioning. Pozza et al.^13^ found that automatic picking could be uniquely predicted by borderline and avoidant traits, while focused picking was associated with a lack of emotion regulation, and mixed picking with sadistic traits. In regard to cognitive processes, focused picking has been compared to focused hair pulling in patients with trichotillomania and has been hypothesized to be a mechanism to reduce negative internal states (urges/thoughts) or emotions (anxiety/depression).^14^ Conversely, automatic picking has been proposed to be a form of habitual self-stimulation.^15^ However, when skin picking disorder subtypes were compared using MRI imaging, there were no structural differences found.^16^

The subtypes of skin picking thus remain a highly understudied topic, and very few studies have examined clinical and neurocognitive differences between the focused, automatic, and mixed subtypes in the same sample of people with skin picking disorder. Understanding these differences may provide a greater understanding of the skin picking disorder subtypes and may have implications for improved treatments. Thus, the goal of the present study was to analyze differences in symptom severity, impulsivity, emotional regulation, cognitive performance, and presence of comorbid psychiatric conditions between the focused, automatic, and mixed subtypes in the same sample of adults with skin picking disorder. We hypothesized that focused pickers would have greater symptom severity, impulsivity, and comorbidities and lower emotional regulation and cognitive performance than automatic or mixed pickers.

## Methods

This study enrolled 83 adults aged 18–65 with a current DSM-5 diagnosis of skin picking disorder over a time period from March 2017 to April 2018. Participants were recruited using social media, advertisements, and flyers and underwent an in-person assessment at the University of Chicago. Inclusion criteria were (1) DSM-5 diagnosis of skin picking disorder, (2) aged 18–65 years, (3) fluency in English, and (4) capable of providing informed consent. Participants were excluded if they were unable to give informed consent or unable to understand the study procedures. The study and consent statement were approved by the Institutional Review Board of the University of Chicago (IRB16-0739). After receiving a complete description of the study, participants provided written informed consent. The authors assert that all procedures contributing to this work comply with the ethical standards of the relevant national and institutional committees on human experimentation and with the Helsinki Declaration of 1975, as revised in 2008.

## Assessments

Demographic data and information regarding current psychotherapy and/or psychiatric medications were collected through a self-reported assessment completed by participants. Afterwards, each participant underwent a semi-structured interview to acquire data regarding clinical characteristics of skin picking disorder. We used the skin picking disorder module from the Minnesota Impulse Disorders Interview (MIDI)^17^ to confirm the diagnosis of skin picking disorder. We used the Mini-International Neuropsychiatric Interview 7.0^18^ to diagnose co-occurring psychiatric disorders. Additionally, participants completed the following clinical and neurocognitive assessments:

1. Milwaukee Inventory for the Dimension of Adult Skin Picking (MIDAS)—A 12-item self-reported questionnaire designed to assess the extent of “focused” (conscious, intentional) and “automatic” (unintentional, not within awareness) domains of skin picking by providing a numeric score for each with a range of 6–30. Higher scores indicate a stronger association with the given domain.^11^
2. Barratt Impulsiveness Scale (BIS)—A 30-question self-reported questionnaire designed for the assessment of impulsivity. It measures 6 first-order domains (attention, motor, self-control, cognitive complexity, perseverance, and cognitive instability impulsiveness) and 3 second-order domains (motor, non-planning, and attentional impulsiveness).^19^ The BIS reported moderate internal consistency in this study (α =0.60).
3. Emotion Regulation Questionnaire (ERQ)—A 10-item self-report scale designed to measure respondents’ tendency to regulate their emotions in two domains: cognitive reappraisal and expressive suppression.^20^ Respondents answer each item on a 7-point Likert-type scale ranging from 1 (strongly disagree) to 7 (strongly agree). The ERQ demonstrated good internal consistency in this study (α =0.87 for cognitive reappraisal, α =0.75 for expressive suppression).
4. Skin Picking Scale Revised (SPS-R)—A 8-item self-report scale measuring skin picking disorder severity in two domains: impairment and symptom severity.^21^ Higher scores indicate greater impairment or symptom severity. Both factors demonstrated excellent internal consistency in this study (α =0.93 for impairment, α =0.96 for symptom severity).
5. Intra-Extra Dimensional Shift (IED)—A neurocognitive assessment measuring cognitive flexibility that is set-shifting, where subjects attempt to learn an underlying rule about which of two stimuli presented on the computer screen is correct. After making each choice by touching the stimulus, feedback is given (“correct” or “incorrect” appeared on the screen). Through trial and error, participants learn the underlying rule. Over the course of the task, the rule is changed by the computer to assess different components of flexible responding. A higher number of total errors in the assessment **indicates a greater degree of impairment in cognitive flexibility, specifically reflecting difficulties in shifting attentional set between stimulus dimensions and/or deficits in learning from feedback.** The key outcome measure is the total number of errors on the assessment by the participant.^22^
6. Cambridge Gambling Task (CGT)—A neurocognitive assessment measuring decision-making. For each trial, 10 boxes are shown, some blue and some red, with a token having been hidden behind one of these. The participant selects the color of box they believe a token is hidden behind, then decides how many points they want to gamble that they made the right decision. The main measures of decision-making on the task are the proportion of points gambled overall, the proportion of rational decisions made (where the subject chooses a color in the majority), and the extent of risk adjustment (extent that subjects change the amount gambled based on new probabilities shown).^23^
7. Stop Signal task (SST)—A neurocognitive assessment measuring motor inhibition. Subjects are instructed to view and respond to left or right facing arrows that appear on a computer screen one at a time in a rapid fashion.^24^ For example, if a left arrow appears, they press a left button, and vice versa for right facing arrows. When an auditory stop-signal “beep” occurs, participants attempt to withhold their motor response for the given trial. The SST has been shown to be a representative proxy for the speed of the inhibitory process, and specifically, the latter half of the task reflects the participant’s ability of sustained inhibitory control.^25^ The main outcome measure on the task is the stop-signal reaction time (in milliseconds) for the last half of the task.

## Statistical analysis

All statistical analysis was performed using SPSS v29. To identify skin picking disorder groups, we used the 12-item MIDAS. For each participant, the MIDAS provided a “focused” score and an “automatic” score. We then used K-means clustering to identify 4 specific subtype “clusters” into which participants would be categorized. K-means clustering is a data-driven statistical method that fits a certain number of centroids and assigns each datapoint (in this study, represented by a participant’s MIDAS focused and automatic scores) to the best fitting cluster. A datapoint is assigned to a particular cluster if it is closer to that cluster’s centroid than any other centroid. In other words, it attempts to individually fit each datapoint to an assigned cluster that is separated across multi-dimensional space. Based on existing literature,^26^ we used K-means clustering to identify 4 distinct groups that subjects were placed in based on their MIDAS scores and assigned each to a specific subtype of skin picking disorder: high focused/high automatic (mixed subtype); high focused/low automatic (focused subtype); low focused/high automatic (automatic subtype); low focused/low automatic (low symptom subtype). The cluster groups were then assessed for between-group differences in clinical and neurocognitive outcomes using ANOVA or Chi-Squared tests (*p* < 0.05). Demographics and comorbid psychiatric diagnoses were presented as frequencies. Variables assessing the clinical and neurocognitive outcomes were calculated as mean scores accompanied by standard deviations. Effect size was measured using η^2^ (eta-squared) for ANOVA tests and Cramer’s V for chi-squared tests. The level of significance for all statistical tests was set at 0.008 after using a Bonferroni correction for multiple comparisons.

## Results

**Figure 1.**
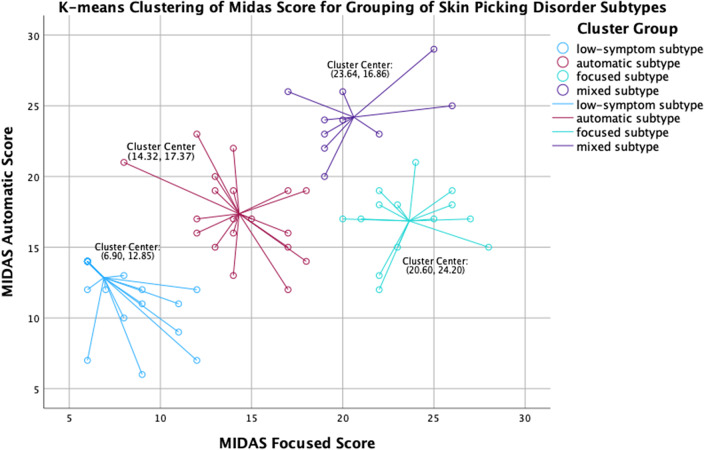
K-means clustering of midas score for grouping of skin picking disorder subtypes.

A total of 83 adults with DSM-5 with skin picking disorder (mean age = 30.1 [SD 9.1]; 89.2% females) were enrolled. The centroid points for each of the 4 cluster groups obtained from K-means clustering of MIDAS scores are listed in Table 1, and a 2D scatter plot of the cluster groups is shown in Figure 1. A Silhouette Cluster Analysis was conducted to evaluate the quality and appropriateness of the 4 cluster groups. The results showed a total mean silhouette score of 0.80, indicating a strong level of cohesion and separation between the 4 cluster groups. Of the 83 adults, 40 (48.2%) were classified as low focused-low automatic, 19 (22.3%) low focused-high automatic, 14 (16.9%) high focused-low automatic, and 10 (12.0%) high focused-high automatic. The mean “focused” and “automatic” MIDAS subscores for all subjects were 18.93 (SD = 5.15) and 17.09 (SD = 4.60), respectively. Demographic characteristics of the 4 subtypes are presented in Table 2, along with results from the 3 clinical outcome measures: BIS, ERQ, and SPS-R.

**Table 1.** Cluster Centers Generated from K-Means Clustering of MIDAS Responses

Cluster	MIDAS Focused Score	MIDAS Automatic Score
1	6.90	12.85
2	14.32	17.37
3	23.64	16.86
4	20.60	24.20

*Note:* Cluster 1 = low focused-low automatic (low-symptom subtype); Cluster 2 = low focused-high automatic (automatic subtype); Cluster 3 = high focused-low automatic (focused subtype); Cluster 4 = high focused-high automatic (mixed subtype).

**Table 2.** Demographic and Clinical Variables of Group Clusters

		1		2		3		4		Statistic	df	η^2^ or Cramer’s V value	Significance (*p*-value)	Significant Cluster Group Comparisons
		n	M (SD)	n	M (SD)	n	M (SD)	n	M (SD)					
Age (years)		40	29.35 (7.60)	19	31.26 (7.64)	14	32.14 (13.34)	10	27.70 (8.25)	0.694f	3,79	0.026	0.559	
Race [%]	Caucasian	33	[82.5]	17	[89.5]	12	[85.8]	5	[50.0]	13.044c	9	0.229	0.161	
	Asian	3	[7.5]	0	[0]	1	[7.1]	1	[10.0]					
	Biracial	1	[2.5]	2	[10.5]	1	[7.1]	3	[30.0]					
	Other	3	[7.5]	0	[0]	0	[0]	1	[10.0]					
Ethnicity [%]	Hispanic	32	[84.2]	17	[94.4]	13	[92.9]	7	[70.0]	3.894c	3	0.221	0.273	
	Non-Hispanic	6	[15.8]	1	[5.6]	1	[7.1]	3	[30.0]					
Sex [%]	Female	36	[90.0]	15	[78.9]	13	[92.9]	10	[100.0]	3.492c	3	0.205	0.322	
	Male	4	[10.0]	4	[21.1]	1	[7.1]	0	[0]					
BIS		39	60.08 (11.41)	19	60.21 (11.60)	14	64.86 (9.48)	9	62.11 (12.97)	0.681f	3,77	0.094	0.566	
ERQ	Reappraisal	39	27.90 (7.60)	19	27.26 (5.48)	14	23.07 (7.42)	8	27.88 (8.27)	1.639f	3,76	0.157	0.187	
	Suppression	40	12.05 (5.33)	18	12.78 (5.35)	14	14.21 (4.35)	9	13.11 (3.10)	0.677f	3,77	0.093	0.569	
SPSR	Severity	40	1.28 (2.07)	19	7.47 (2.41)	14	9.36 (2.59)	10	9.00 (3.50)	61.476f	3,79	0.764	<0.001*	**1 versus 2** **1 versus 3** **1 versus 4**
	Impairment	39	0.85 (1.80)	19	4.42 (2.34)	14	8.21 (3.14)	9	5.22 (2.91)	38.620f	3,77	0.684	<0.001*	**1 versus 2** **1 versus 3** **1 vs 4** **2 versus 3**

*Note:* 1 = Low focused-low automatic; 2 = Low focused-high automatic; 3 = high focused-low automatic 4 = high focused-high automatic (according to MIDAS: Milwaukee Inventory for the Dimensions of Adult Skin Picking). All results are mean (SD) unless otherwise noted.

Statistic: c = Chi-square; f = F ratio.

η2 = eta-squared.

*indicates significance at *p* < 0.008 (Bonferroni Correction applied).

Abbreviations: BIS, Barratt Impulsiveness Scale; ERQ, Emotion Regulation Questionnaire; SPSR,Skin Picking Scale Revised.

The subtypes did not differ significantly in age, race/ethnicity, sex, BIS, or ERQ scores. When compared to the low-symptom subtype, each of the other groups had significantly higher skin picking severity and impairment. There were no significant differences in skin picking symptom severity between the focused, automatic, or mixed subtypes. The focused subtype had significantly greater impairment scores compared to the automatic subtype. Table 3 shows the number and percentages of co-occurring psychiatric disorders in each cluster group. Rates of co-occurring disorders did not differ between groups. In terms of neurocognitive assessments, there were no statistically significant differences between cluster groups on any of the tasks, as shown in Table 4.

**Table 3.** Comorbid Psychiatric Conditions of Group Clusters

		1		2		3		4		Statistic	df	Cramer’s V value	Significance (*p*-value)
		n	%	n	%	n	%	n	%				
Major depressive disorder	Yes	7	17.9	8	42.1	8	57.1	4	40.0	8.630c	3	0.324	0.0418
	No	32	82.1	11	57.9	6	42.9	6	60.0				
Generalized anxiety disorder	Yes	6	15.4	5	26.3	6	46.2	4	40.0	6.059c	3	0.274	0.109
	No	33	84.6	14	73.6	7	53.8	6	60.0				
OCD	Yes	2	5.1	0	0.0	3	21.4	1	10.0	5.993c	3	0.270	0.112
	No	37	94.9	19	100.0	11	78.6	9	90.0				
Body dysmorphic disorder	Yes	0	0.0	1	5.3	2	20.0	0	0.0	7.447c	3	0.307	0.059
	No	38	100.0	18	94.7	10	100.0	10	100.0				
Social anxiety disorder	Yes	1	2.6	2	10.5	2	20.0	1	10.0	3.164c	3	0.199	0.367
	No	38	97.4	17	89.5	10	100.0	9	90.0				
PTSD	Yes	2	5.1	2	10.5	2	20.0	0	0.0	2.831c	3	0.188	0.418
	No	37	94.9	17	89.5	10	100.0	10	100.0				
ADHD	Yes	1	2.6	1	5.3	4	33.3	1	10.0	11.261c	3	0.375	0.0138
	No	38	97.4	18	94.7	8	66.7	9	90.0				
Substance use disorder	Yes	0	0.0	1	5.3	0	0.0	0	0.0	3.251c	3	0.202	0.354
	No	39	100.0	18	94.7	12	100.0	10	100.0				
Trichotillomania	Yes	18	46.2	5	26.3	3	25.0	2	20.0	4.279c	3	0.231	0.233
	No	21	53.8	14	73.6	9	75.0	8	80.0				

*Note:* 1 = Low focused-low automatic; 2 = Low focused-high automatic; 3 = high focused-low automatic. Cluster 4 = high focused-high automatic (according to MIDAS: Milwaukee Inventory for the Dimensions of Adult Skin Picking). All results are n (%) unless otherwise noted. c = Chi-Squared.

Abbreviations: OCD, Obsessive Compulsive Disorder; PTSD, Post-Traumatic Stress Disorder; ADHD, Attention Deficit Hyperactivity Disorder.

**Table 4.** Neurocognitive Variables of Group Clusters

		1		2		3		4		Statistic	df	η^2^ value	Significance (*p*-value)
		n	M (SD)	n	M (SD)	n	M (SD)	n	M (SD)				
IED total errors		39	29.74 (22.69)	19	19.37 (20.03)	13	24.92 (19.83)	10	23.10 (19.49)	1.084f	3,77	0.123	0.361
CGT	Delay aversion	38	0.29 (0.24)	19	0.25 (0.22)	13	0.31 (0.16)	10	0.11 (0.41)	1.512f	3,76	0.150	0.218
	Overall bet proportion	39	0.55 (0.12)	19	0.56 (0.13)	13	0.58 (0.18)	10	0.48 (0.15)	0.979f	3,77	0.116	0.407
	Quality of decision making	39	0.96 (0.09)	19	0.97 (0.06)	13	0.94 (0.09)	10	0.96 (0.05)	0.296f	3,77	0.054	0.828
	Risk adjustment	39	1.29 (1.04)	19	1.58 (1.12)	13	1.05 (0.78)	10	0.98 (0.85)	1.075f	3,77	0.122	0.365
	Deliberation time	39	1743.57 (735.61)	19	1753 (464.49)	13	1799.29 (674.92)	10	1822.02 (428.73)	0.057f	3,77	0.00	0.982
SST SSRT (last half)		39	229.15 (103.70)	19	219.86 (34.98)	11	267.75 (215.41)	10	232.16 (110.01)	0.467f	3,75	0.076	0.706

1 = Low focused-low automatic; 2 = Low focused-high automatic; 3 = high focused-low automatic.

Cluster 4 = high focused-high automatic (according to MIDAS: Milwaukee Inventory for the Dimensions of Adult Skin Picking).

All results are mean (SD) unless otherwise noted.

f = F ratio.

η^2^ = eta-squared.

Abbreviations: IED, Intra-Extra Dimensional Set Shift; CGT, Cambridge Gambling Task; SST SSRT, Stop Signal Task – Stop Signal Reaction Time.

## Discussion

This study adds to the limited literature examining phenomenological subtypes of skin picking disorder and is the first to analyze differences in skin picking disorder symptom severity, impulsivity, and presence of comorbidities between the focused, automatic, and mixed subtypes. The results showed that there were no differences in symptom severity between the focused and the automatic or mixed group, which was contrary to our hypothesis that focused picking would have greater symptom severity. Furthermore, the groups did not differ in neurocognitive measures. Therefore, these findings suggest that the idea of automatic and focused picking may not be particularly helpful in subtyping skin picking disorder. Although the distinction between automatic and focused skin picking may have some clinical relevance, there were no coherent subtypes of the disorder based on these variables. This may be unsurprising as both focused and automatic picking may characterize the same picking episode, or certainly the same person across episodes.^12^ ^,^^27^ In the case of trichotillomania, assessments have recently begun to examine intentional and emotional types of pulling instead of focused and automatic.^28^ Whether this nuanced recategorization of subtypes would be useful in the case of skin picking disorder remains to be seen.

If both high focused and high automatic pulling are associated with greater symptom severity, then these findings may highlight why many people with skin picking disorder do not respond to most available psychotherapy treatments. In the case of trichotillomania (a disorder closely related to skin picking disorder), habit reversal therapy may work best for focused behavior.^29^ Strengthening the “awareness training” component of habit reversal therapy, or augmenting with more stimulus control, could possibly improve the ability to target automatic picking in conjunction with focused picking.

This study has several limitations that should be considered. First, while the MIDAS has been shown to have strong validity and reliability in past studies,^11^ ^,^^13^ ^,^^30^ it may not be the most accurate or reliable means to differentiate subtypes. Second, we utilized K-means clustering in this study to separate each participant into a given subtype based on their MIDAS scores’ proximity to a specific cluster center. This led to cluster groups that were relatively uneven in the number of participants per cluster, which could have affected the statistical validity of the data. Future work could examine the use of more recent Intention and Emotion subscores as a means of subtyping the disorder. Finally, the largely null findings in this study may be attributed to the use of the Bonferroni correction, which decreases the power of the study and may lead to a higher risk of a type II error.

## Conclusion

Skin Picking Disorder has historically been characterized by different phenomenological subtypes. The findings in this study suggest that focused, automatic, and mixed subtypes do not seem to differ in symptom severity, neurocognitive measures, or presence of comorbid psychiatric disorders. We propose that future studies of skin picking disorder revisit whether subtypes based on focused vs automatic picking have statistical validity and clinical utility. To properly define reproducible subtypes, it is likely going to be necessary to capture a comprehensive range of measurement domains. Clarification of subtypes is likely to require longitudinal data collection since subtypes may only be apparent over time. It would also be interesting to explore subtypes in terms of response to different treatment modalities for skin picking disorder.

## Data Availability

Data available upon reasonable request.
